# An adaptive coordination control solution to boost frequency stability for a hybrid distributed generation system

**DOI:** 10.1371/journal.pone.0321657

**Published:** 2025-05-13

**Authors:** Hossam S. Salama, Gaber Magdy, Abualkasim Bakeer, Thamer A. H. Alghamdi, Mohammed Alenezi, Mahmoud Rihan

**Affiliations:** 1 Electrical Engineering Department, Faculty of Engineering, Aswan University, Aswan, Egypt; 2 Electrical Engineering Department, Faculty of Energy Engineering, Aswan University, Aswan, Egypt; 3 Electrical Engineering Department, Faculty of Engineering, Al-Baha University, Al-Baha, Saudi Arabia; 4 Wolfson Centre for Magnetics, School of Engineering, Cardiff University, Cardiff, United Kingdom; 5 Electrical Engineering Department, Faculty of Engineering, South Valley University, Qena Egypt; Aalto University, FINLAND

## Abstract

Under the current global circumstances, the urgent need to exploit renewable energy sources (RESs) is increasing. Increased penetration of RESs in hybrid distributed generation systems (HDGSs) poses a challenge due to the unsettled nature of RESs on frequency stability (FS). So, coordination between RESs is essential to sustaining FS. To boost FS in HDGSs, this study presents an adaptive coordination control (ACC) solution regarding RESs, a fuel cell (FC)-based energy storage system (ESS), and an aqua electrolyzer (AE) for producing hydrogen. In the studied system, the surplus energy of RESs is employed to supply electrolysis by AE to store hydrogen energy inside FC. The proposed ACC solution employs fuzzy control to dynamically adjust the RES energy ratio allocated to AE (the surplus energy “K_n_” is a fraction of the overall RES-generated energy). The studied HDGS includes photovoltaic (PV) plants, wind turbines (WT), AE coupled with FCs, ESSs (e.g., flywheels and batteries), and diesel generators (DG). The suggested solution changes K_n_ according to the frequency deviations to maximize the benefits of RESs alongside damping frequency variations. This study examined the proposed solution under different loading situations and compared it to previous research that took K_n_ as a fixed value.

## 1. Introduction

The increased global energy demand, besides the world’s conflicts and epidemics, leads to increasing the prices of fossil fuels, as they are in limited and restricted amounts, causing a repeated multiplying of the electricity tariffs and raising the emissions of greenhouse gases (GHG) [1]. This necessitated resorting to renewable energy sources to meet the requirements of sustainable development for a safer future [2–4]. Traditional central power systems (CPSs) rely primarily on fossil fuels and have long transmission and distribution networks. So, these grids have huge losses, which reduce their overall efficiency. They are also complex due to their huge size, requiring extensive upkeep, which weakens the reliability and security of these networks. Due to the participation of renewable energy sources (RESs) and the absence of long transmission networks, hybrid distributed generation systems (HDGSs) provide a costless alternative solution with more reliability and efficiency, better quality, and higher sustainability than traditional CPSs [5–8]. However, one of the most prominent challenges facing RESs is the uncertainty of these resources due to their interrupted nature. This is reflected in the power produced from those sources, which would fluctuate frequently. These frequency fluctuations are in addition to the frequency fluctuations that already exist in the network due to the changing nature of the loads, leading to increased frequency instability [9–11]. This necessitated that HDGSs include robust load frequency control (LFC) systems.

### 1.1. Literature overview

In the literature, various control solutions have been proposed to improve FS in HDGSs, some utilize optimization algorithms (OAs) to fine-tune the controller parameters optimally. In [10], a fractional-order proportional–integral–derivative–accelerator with a low-pass filter (FOPIDA-LPF) with grey wolf optimization (GWO) was employed. In [11], Ultra-Local Model (ULM) control, based on the African Vultures OA, was included in the original secondary frequency regulation loop (i.e., integral control). An interval type-2 fuzzy-PID, based on a modified equilibrium optimizer, is presented in [12]. In [13], relying on a harmony search OA, a feedforward fractional-order PID (FFOPID) is utilized. Whereas a hybrid fuzzy proportional derivative-tilt integral derivative (FPD-TID) controller relying on a chaotic crow search algorithm (CCSA) is presented in [14]. In [15], a PD-based adaptive differential evolution method with filter plus (1+PI) is proposed. A proportional derivative-proportional integral derivative (PD-PID) controller relying on a black hole OA was adopted in [16]. A hybrid fuzzy PD-PI controller with A modified moth swarm algorithm (mMSA) was employed [17]. In [18], a hybrid control solution was suggested, including distributed leader-following consensus and model predictive control. A PID controller with a derivative filter relying on a bacterial foraging OA was used in reference [19]. The PI, in addition to the clegg integrator (CI) reset control method, was suggested in [20] for secondary frequency control (SFC). Salp Swarm OA was used with a cascaded PI-PD controller for the LFC of a microgrid (µG) that incorporates electric vehicles (EVs) [21]. A fractional-order (FO)-fuzzy-PID controller was discussed in [22].

In [23,24], energy storage solutions (ESSs) were used with the RESs to uphold FS. Superconducting magnetic energy storage (SMES) was employed with a PID and droop controller to support the µG with adequate power during abrupt load variations, enhancing the FS [25]. Two main control strategies were adopted in [26]. The first works to save the balance between load demand and generation, as it works in unbalancing cases due to the variable nature of RESs; in those cases, the controller injects real power from the batteries into the grid, while the second one works to suppress frequency fluctuations and makes up for the system’s low inertia. The suggested control strategy in [27] adopted power sharing between the battery energy storage system (BESS) and fuel cell (FC) via an adaptive droop control. In [28], to attain SFC, the PID controller gains are used as inputs, together with the integral time absolute error (ITAE), to a control mechanism that relies on the GWO. To get the ideal membership function (MF) values for saving the FS, hybrid OAs, including both the GWO and particle swarm optimization (PSO), were applied in conjunction with adaptive fuzzy logic control relying on a PI controller [29].

In [30], the coordination control strategy (CCS) between RESs and FC was given, utilizing an adaptive fuzzy PID controller and relying on a simplified GWO form for the LFC of an HDGS. An investigation of HDGS, constructed from wind, PV systems, and FCs, was conducted in Iraq (Bahr al-Najaf) [31].

Since hydrogen production units (HPUs) have long-term power absorption and large capacities, these units can be utilized to bridge the extensive power gaps in the circumstances of significant RES adoption. In [32], considerable employment of HPUs was introduced, and the adopted CCS included two main stages. The strategy was based on maintaining the maximum possible generation of the PV systems. Then, the first stage of the CCS was made to save the optimal energy conversion efficiency (from electrical to hydrogen) of the HPU—the second stage aimed to regulate the instantaneous power and charge storage state.

To have the lowest generation costs and meet the demands, the EVs and the RES plants combined as virtual stations, where the suggested control system relied on lifelong learning through three stages [33]. The initial one represented lifelong learning, and the next stage aimed to determine the generation expenses of the available reserve resources and then sort the costs of the available scenarios to meet the demands in ascending order. The last stage had the CCS with an online optimization, and the EV charging demands were considered.

A super-twisting algorithm (STA) technique was presented in [34] to decrease the voltage fluctuations of the buses and enhance all the rationality of power distribution and current circulation. On the other hand, the power-sharing percentage (PSP) between RESs and FC was not studied, nor were the fluctuations of the loads and wind system examined.

For effective utilization of electrolysis and FC, a two-time-scale energy management system (EMS) was provided [35]. However, the suggested approach did not consider the high load variation or the PSP. Although [36] has an adopted EMS, the applied control technique did not consider the PSP between FC and RES.

An integral controller combined with a linear quadratic regulator was employed in [37] to regulate the FS of a µG. Nevertheless, the PSP was not considered, and the suggested method’s efficacy was verified under mild load perturbations.

The decentralized coordination control technique between generation, HPU, ESS, and FC in DC and AC µGs was introduced in [38] and [32]. Nevertheless, neither study addressed the PSP or the FC electrical characteristics.

In [39], a stochastic model was suggested for the CCS of RESs; however, protections for state-of-charge (SoC) and overpower were not studied. Furthermore, the PSP was not considered in the decentralization technique. Recently, federated learning (FL) integrated with model-agnostic meta-learning (MAML) has emerged as a promising approach for short-term load forecasting (STLF), addressing both data privacy concerns and model personalization challenges. For instance, a federated model-agnostic meta-learning (FMAML)--based method was proposed to improve STLF accuracy for distribution transformer supply zones while preserving data privacy. This approach leverages MAML to create personalized forecasting models for individual clients, significantly enhancing the adaptability and compatibility of federated learning. Additionally, the stochastic controlled averaging (SCA) algorithm was introduced as an aggregation method to mitigate the client-drift phenomenon, which can cause slow convergence or even divergence during training, especially under highly heterogeneous data conditions. Numerical results confirmed that the FMAML method achieves superior forecasting accuracy and robustness compared to existing processes, particularly in data heterogeneity and packet dropout scenarios. Future developments in this area aim to refine the FMAML framework further, providing customized forecasting models tailored to individual clients to enhance adaptability and prediction accuracy [40].

Recent advancements have highlighted the importance of resilient control strategies for load frequency control (LFC) in microgrids (MGs) with significant wind energy integration, especially considering practical challenges such as phasor measurement unit (PMU) faults and communication intermittency. A hierarchical control architecture employing model predictive control (MPC) combined with an intensified event-triggered scheme (ETS) was proposed to enhance computational efficiency and tracking accuracy for wind power integration. Robustness specifications were addressed by introducing uncertain matrices into conventional small-signal LFC models, accommodating parameter variations resulting from fluctuating wind power outputs. The effectiveness and resilience of this approach were validated through hardware-in-the-loop (HIL) experiments, demonstrating superior frequency regulation performance compared to traditional controllers under varying probabilities of PMU failures and intermittent communication scenarios. Future research directions include systematically analyzing historical data to accurately estimate failure probabilities, further enhancing the reliability and robustness of resilient LFC solutions [41]. Table 1 presents a comparison of the most pertinent studies.

**Table 1:** A synopsis of the most recent studies.

Ref.	Year	Contribution	Control strategy	Incompetence
[10]	2023	LFC	FOPIDA-LPF with GWO	PSP (between RESs & FC) Coordination, Considering the electrical characteristics of FC
[12]	2021	LFC	Type-2 fuzzy PID with modified EO	PSP (between RESs & FC) Coordination, Considering the electrical characteristics of FC
[34]	2021	Decrease the voltage fluctuations and enhance all the rationality of power distribution and current circulation.	STA	PSP (between RESs & FC) Coordination, High fluctuations of the loads were not examined, and the wind system was not included.
[36]	2020	EMS considering the electrical characteristics of the studied µG components	*p-f* droop control	PSP (between RESs & FC) Coordination,
[37]	2020	LFC	Integral controller combined with a linear quadratic regulator	PSP (between RESs & FC) Coordination, High fluctuations of the loads were not examined
[38,39]	2020	Decentralized coordination control	Effective adaptive control	PSP (between RESs & FC) Coordination, Considering the electrical characteristics of FC
[35]	2019	EMS considering the electrical characteristics of the FC and electrolyzer	Two-time-scale EMS	PSP (between RESs & FC) Coordination, High fluctuations of the loads were not examined
[40]	2017	Systematic coordination of RESs.	Stochastic model	PSP (between RESs & FC) Coordination, SoC protection, Overpower protection
[41]	2025	Improve STLF accuracy for distribution transformer supply zones while preserving data privacy	federated learning (FL) integrated with model-agnostic meta-learning (MAML)	Refine the FMAML framework further, providing customized forecasting models tailored to individual clients to enhance adaptability and prediction accuracy
[42]	2025	load frequency control (LFC) in microgrids (MGs) with significant wind energy integration and enhanced computational efficiency and tracking accuracy for wind power integration.	MPC combined with an intensified event-triggered scheme (ETS)	Include systematically analyzing historical data to accurately estimate failure probabilities, further enhancing the reliability and robustness of resilient LFC solutions

Table 1 highlights the issues that were not treated in previous studies and can be summarized as follows:

- PSP (between RESs & FC) Coordination,
- Considering the electrical characteristics of FC,
- Examining the reliability of the system under high fluctuations in the loads,
- Decentralized strategy,
- Overpower protection,
- SoC protection.

Also, in the previously mentioned studies, the PSP (between RESs and FC) was either not considered or considered with a fixed value.

### 1.2. Main contributions

Mainly, this study introduces an adaptive CCS to effectively govern the value of the PSP between the FC and the RESs (K_n_) via a PI controller. The suggested methodology relies on adjusting the K_n_ based on a fuzzy logic controller. To ensure the FS of the studied system is saved, the proposed study considers the various loading conditions and uncertainties of RESs.

Table 2 shows the main differences between the suggested study and the above-mentioned previous studies. A performance comparison between the proposed methodology and previous methods has been introduced to assess the suggested CCS’s efficacy.

**Table 2:** The main differences between the suggested work and the above-reviewed studies.

Ref.	Fluctuations			FC	AE	Consideration of K_n_	K_n_ Tunning	Key features	Control Strategy
	Load	RESs							
[11]	High		Wind + PV	Χ	Χ	Χ	N/A	LFC	ULM control, based on AVOA, was included in integral control (the original secondary frequency regulation loop)
[13]	High		Wind + PV	Χ	Χ	Χ	N/A	LFC	FFOPID with harmony search OA
[14]	Low		Wind + PV	√	√	Constant	N/A	LFC	FPD-TID with CCSA
[17]	Low		Wind + PV	√	√	Constant	N/A	LFC	Hybrid fuzzy PD-PI controller with Mmsa
[19]	Low		Wind	√	√	Χ	N/A	LFC	PID with a derivative filter relying on a bacterial foraging OA
**Suggested work**	High		Wind + PV	√	√	Variable (Adaptively)	Fuzzy logic Controller	Adaptive CCS, LFC, For the adaptation with frequency deviations, the suggested adaptive CSS effectively adjusts the K_n_.	PID utilizes a fuzzy logic controller to adjust Kn adaptively.

The remainder of this essay is organized as follows: Section 2 displays the studied HDGS model. Whereas the suggested adaptive CCS is covered in Section 3, Section 4 presents the simulation findings. Lastly, the study is concluded in Section 5.

## 2. Studied system

The studied HDGS includes various systems: diesel generators (DG), wind turbines (WTs), PV, AE, FCs, BESS, flywheel energy storage systems (FESS), and EVs.

**Fig 1:**
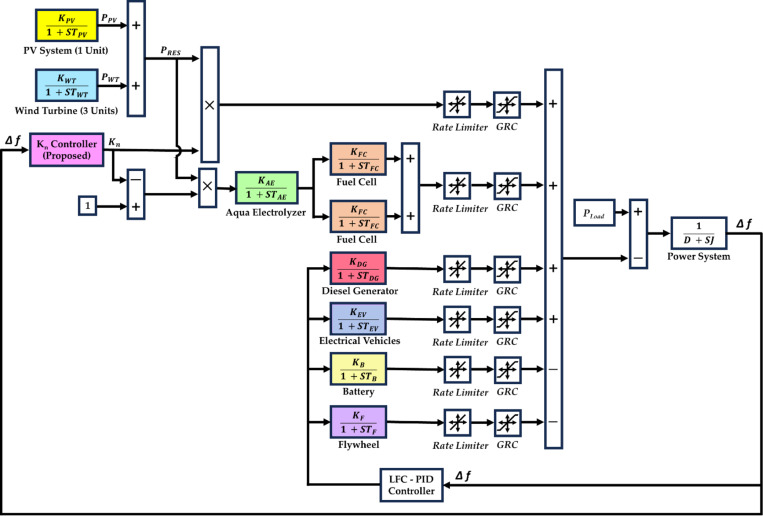
The studied HDGS, including the proposed CCS.

Transfer function (TF) models are used to represent each of these systems, as shown in Fig 1. The gain (k) values and time constants are provided in Table 3 [42,43]. A PID controller is applied for load frequency control (LFC), as described in [44].

**Table 3:** The basic control parameters of the studied HDGS.

Element	Gain		Time Constant	
	Symbol	Value	Symbol	Value
PV System	*K*_*PV*_	1	*T*_*PV*_	1.8
Wind Turbine	*K*_*WT*_	1	*T*_*WT*_	1.5
Aqua Electrolyzer	*K*_*AE*_	0.002	*T*_*AE*_	0.5
Fuel Cell	*K*_*FC*_	0.01	*T*_*FC*_	4
Diesel Generator	*K*_*DG*_	0.003	*T*_*DG*_	2
Electric Vehicles	*K*_*EV*_	1	*T*_*EV*_	1
Battery	*K*_*B*_	-0.003	*T*_*B*_	0.1
Flywheel	*K*_*F*_	-0.01	*T*_*F*_	0.1
**Power System**	**Damping Factor (*D*)**		**Virtual Inertia Factor (*J*)**	
	0.3		0.4	

### 2.1. PV System

The PV system converts solar radiation directly into electrical power. The power generated by the PV system (*P*_*PV*_) depends primarily on solar irradiance and ambient temperature. Equation (1) describes the power generated by the PV system. Meanwhile, the simplified TF model of the PV system is described in Equation (2) [42,43].


$$P_{PV}=\;A_{PV}\;\emptyset \;\eta \;[\;1-0.005\;\left(\;T_{a}+25\;\right)$$ (1)

$$TF_{PV}\;\left(S\right)=\;\frac{K_{PV}}{1+S\;T_{PV}}\;=\;\frac{\Delta \;P_{PV}}{\Delta \;\emptyset }$$ (2)

Where:

***A***_***PV***_: Area of PV panels (m²), representing the physical size.

***ϕ***: Solar irradiance (W/m²), indicating solar power intensity.

***η***: Efficiency of PV modules, showing conversion efficiency.

***T***_***a***_: Ambient temperature (°C), affecting module efficiency negatively due to heat losses.

***K***_***PV***_: Steady-state gain, reflecting sensitivity to irradiance variations.

***T***_***PV***_: Time constant (s), representing the system’s dynamic response time to irradiance changes.

### 2.2. Wind turbine system

Three wind turbines are presented in the investigated wind generation system, which generates *P*_*WG*_. Equation (3) clearly describes the obtained mechanical power *P*_*m*_ of the WT [45–47], while Equation (4) represents the TF of the WT system [42,43].


$$P_{m}=\frac{1}{2}\;C_{P}\;\left(\lambda ,\;\beta \right)\;\rho \;A\;V_{wind}^{3}$$ (3)

$$TF_{WT}\;\left(S\right)=\;\frac{K_{WT}}{1+S\;T_{WT}}\;=\;\frac{\Delta \;P_{WT}}{\Delta \;P_{m}}\;$$ (4)

Where:

*C*_*p*_ *(λ,β)* Power coefficient, indicating turbine efficiency dependent on the tip-speed ratio (λ) and blade pitch angle (β).

*ρ*: Air density (kg/m³), physically representing wind mass.

*A*: Rotor swept area (m²), determining wind capture capacity

*V*_*wind*_: Wind speed (m/s), directly influencing power output

*K*_*WT*_: Steady-state gain, representing WT sensitivity to mechanical power changes.

*T*_*WT*_: Time constant (s), reflecting rotor inertia and generator response.

### 2.3. Aqua electrolyzer

The AE depends on utilizing the power from both the PV and WT systems to manufacture the hydrogen, which would then be delivered into an FC-based ESS. Equation (5) states the AE’s TF [42,43].


$$TF_{AE}\;\left(S\right)=\;\frac{K_{AE}}{1+S\;T_{AE}}\;=\;\frac{\Delta \;P_{AE}}{\left(1-K_{n}\right)\;P_{RESs}}\;$$ (5)

Where:

*K*_*AE*_: Conversion factor gain, indicating efficiency and capacity for converting electrical power into hydrogen.

*T*_*AE*_: Electrolyzer time constant (s), representing hydrogen production dynamics.

*K*_*n*_: Fraction of renewable power directed to AE; controls power allocation.

### 2.4. Fuel cells

The studied system is equipped with two identical FCs to maximize efficiency through power sharing and boost system dependability in the event of a failure. Keeping the FC in the system is crucial because it is the primary energy storage that is included to work in tandem with the RESs. Equation (6) presents the FC’s TF [42,43].


$$TF_{FC}\;\left(S\right)=\;\frac{K_{FC}}{1+S\;T_{FC}}\;=\;\frac{\Delta \;P_{FC}}{\Delta \;P_{AE}}$$ (6)

Where:

***K***_***FC***_: FC steady-state gain, indicating power production efficiency from hydrogen.

*T*_*FC*_: Time constant (*s*), representing electrochemical reaction delay in converting hydrogen to electricity.

### 2.5. Diesel generator

The DG can be a stand-alone power source to reduce the imbalance between demand and generation. Equation (7) introduces the DG’s TF [43,44].


$$TF_{DG}\;\left(S\right)=\;\frac{K_{DG}}{1+S\;T_{DG}}\;=\;\frac{\Delta \;P_{DG}}{\Delta \;u_{DG}}$$ (7)

Where:

***K***_***DG***_: Diesel generator gain, reflecting responsiveness to control inputs.

*T*_*DG*_: Time constant (*s*) related to the mechanical inertia and response of diesel engines.

### 2.6. Electric vehicles

Because EVs can function as supplies while discharging and as loads when charging, their performance lessens system fluctuations. Equation (8) states the EV’s TF [42,43].


$$TF_{EV}\;\left(S\right)=\;\frac{K_{EV}}{1+S\;T_{EV}}$$ (8)

Where:

***K***_***EV***_: EV subsystem gain, indicating how rapidly EVs respond to grid commands.

*T*_*EV*_: EV time constant (*s*), representing battery management system response dynamics.

### 2.7. Battery energy storage system

Because it may function as both a load and a power source, the BESS is crucial to maintaining the system’s FS. Equation (9) presents the BESS’s TF [42,43].


$$TF_{B}\;\left(S\right)=\;\frac{K_{B}}{1+S\;T_{B}}$$ (9)

Where:

***K***_***B***_: Battery gain, showing charge/discharge responsiveness.

*T*_*B*_: Battery time constant (s), representing electrochemical and inverter response.

### 2.8. Flywheel energy storage system

The FESS helps maintain the power system’s stability under various operating circumstances. Equation (10) states the FESS’s TF [42,43].


$$TF_{F}\;\left(S\right)=\;\frac{K_{F}}{1+S\;T_{F}}$$ (10)

Where:

***K***_***F***_: Flywheel subsystem gain, indicating rapidity and magnitude of energy exchange.

***T***_***F***_: Flywheel time constant (s), representing rotational inertia dynamics.

### 2.9. Power system model

The input to the power system model is the active power mismatch “ *ΔP* ” (representing the imbalance between demand and generation), while the output is the frequency deviation “ *Δf* ”, as described in Equation (11) [42,43].


$$F_{PS}\;\left(S\right)=\;\frac{1}{D+S\;J}=\;\frac{\Delta \;f}{\Delta \;P}$$ (11)

Where:

***D***: Damping factor, physically representing system damping effects, load sensitivity, and network friction.

*J*: Virtual inertia factor, showing system resistance to frequency changes due to inertia or synthetic inertia contributions.

## 3. The suggested adaptive coordination control strategy

**Fig 2:**
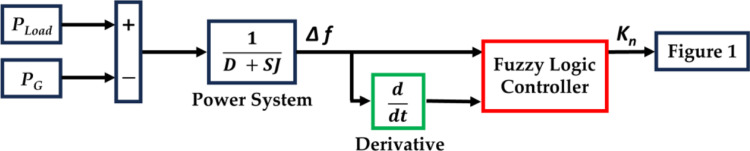
The suggested approach for controlling K_n._

Fig 2 illustrates how the *K*_*n*_ is tuned in the suggested approach. The primary goal of the suggested approach is to accomplish adaptive control for all the RESs, the optimal employing of the AE for hydrogen production, and the best utilization for the FC-ESS, along with boosting the FS of the studied system under various loading scenarios. The suggested approach relies on adjusting the *K*_*n*_ based on a fuzzy logic controller (FLC). FLC has a fuzzification, a fuzzy rationale thinking (knowledge base), a learning base (decision making logic), and a defuzzification.

**Fig 3:**
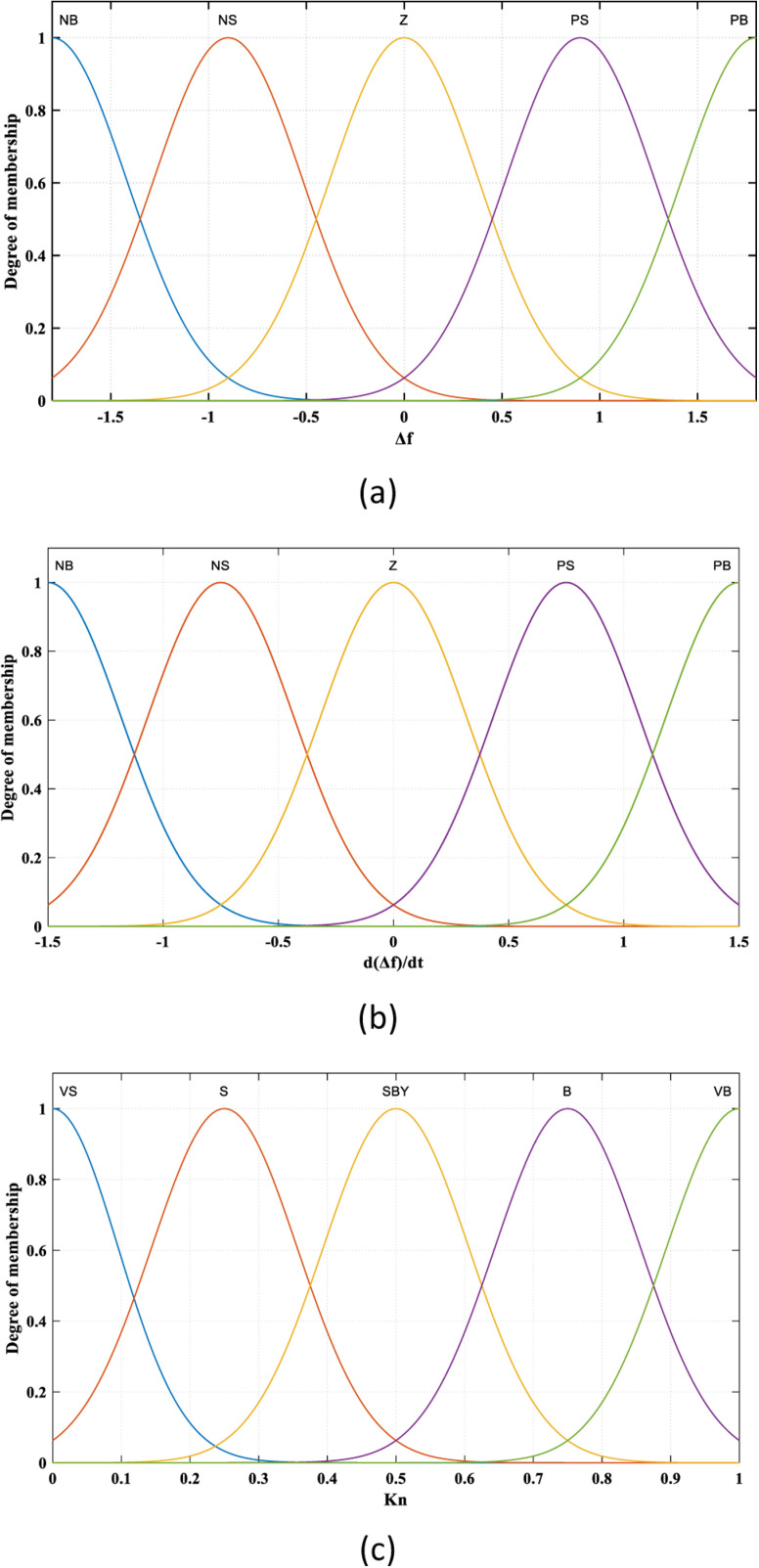
Membership functions of the FLC. (a)The First input “E”, (b) The Second input “CE”, (c) The output “K_n_”.

As shown in Fig 2, FLC has two inputs: the first input is *Δf*, representing the error (E) signal, while the second input represents the change in error (CE). These two inputs are transformed through the fuzzification unit, based on five membership functions (MFs) (Negative Big “NB”, Negative Small “NS”, Zero ”Z”, Positive Small “PS”, and Positive Big “PB”), to a fuzzy number. The output of the FLC is *K*_*n*_, which is transformed based on five MFs (Very Small “VS”, Small “S”, Standby Positive “SBY”, Big “B”, and Very Big “VB”). Sin wave MF is considered in this work for both the “two inputs” and “single output” of the FLC, as illustrated in Fig 3.

**Fig 4:**
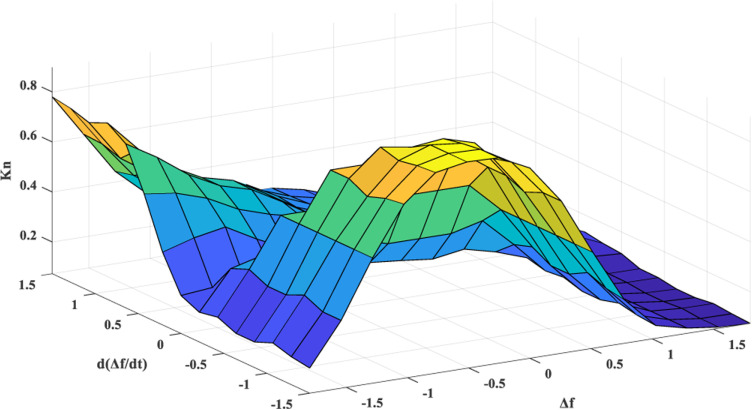
The surface graph view shows the relationship between the inputs and output of the FLC.

**Fig 5:**
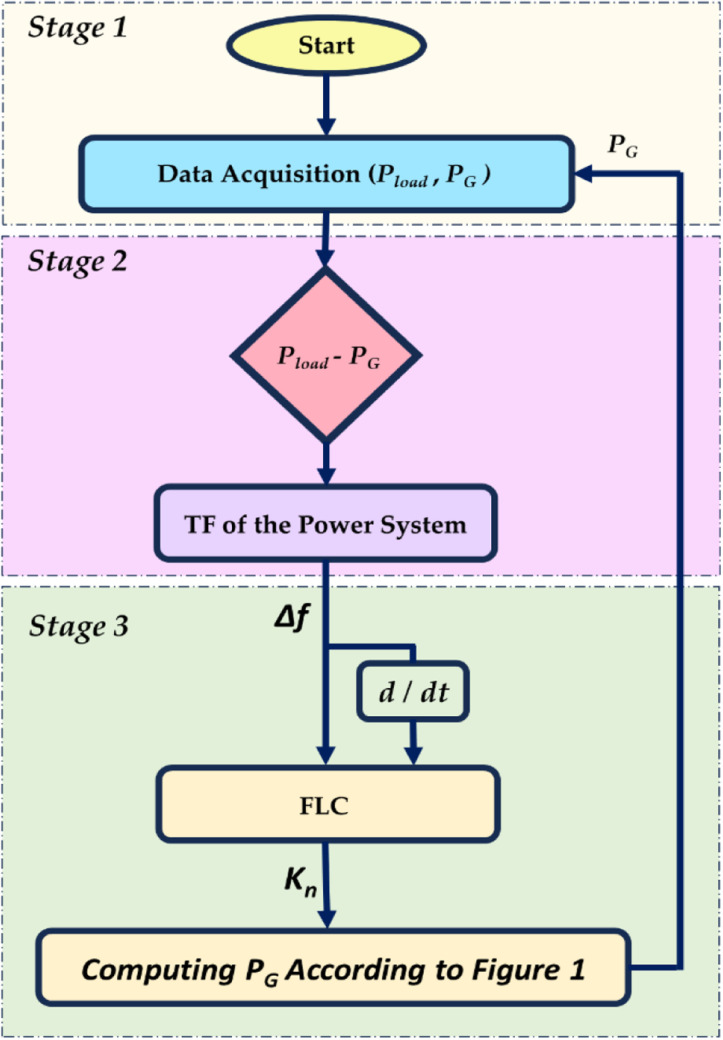
Flowchart of the proposed CCS.

The surface graph, which indicates the relationship between inputs and output, is presented in Fig 4. The flow chart of the proposed adaptive CCS, which aims to improve the FS of HGSs by achieving coordination between RESs, AE, and FC-ESS, is depicted in Fig 5. The structure of the entire suggested adaptive CCS is as follows:

**Stage 1 (Data Acquisition):** Measure, gather, and input the different signals, including the generated active powers, load demand, and the parameters of the FLC, into the adaptive CCS.

**Stage 2 (Observing Frequency Deviation “ *Δf* ”):** computing *Δf* according to the active power difference between the generated power “*P*_*G*_” and the demand “*P*_*Load*_”. The CCS was designed to guarantee that the ” *Δf* “ would not exceed permitted limits. The CCS strategy would define the amount of power supplied into the FC and the AE for hydrogen synthesis and the percentage of total power generation from RES (*K*_*n*_).

**Stage 3 (Defining the best “ *K***_***n***_ **”):** FLC is employed to define the optimal “*K*_*n*_”. Then the obtained “*K*_*n*_” would be used to determine the total “*P*_*G*_”, as illustrated in Fig 1. According to “*K*_*n*_”, only a fraction with a “*K*_*n*_” ratio from the RES would be directed to the AE, while the remaining part would be fed to the HDGS. Thus, the CCS allows for the best possible distribution of FC and RES power, which helps maintain the system frequency within acceptable bounds.

## 4. Results and analysis

Utilizing MATLAB, the examined system depicted in Fig 1 was simulated. To assess the efficacy of the suggested approach, the simulation results, which include four scenarios with various loading situations, were compared to the previous work in [17], which employed a fixed value for the coordination gain of *K*_*n*_.

In this work, the FLC is utilized to optimally define the value of *K*_*n*_, which specifies the injected power into the AE to feed the FC. In the presented scenarios, the cases that adopted the proposed adaptive *K*_*n*_ (based on utilizing the FLC) would be named “Proposed *K*_*n*_ based FLC”. While the other case that adopted constant *K*_*n*_, which would be compared to the proposed strategy, would be called “Constant *K*_*n*_”.

**Fig 6:**
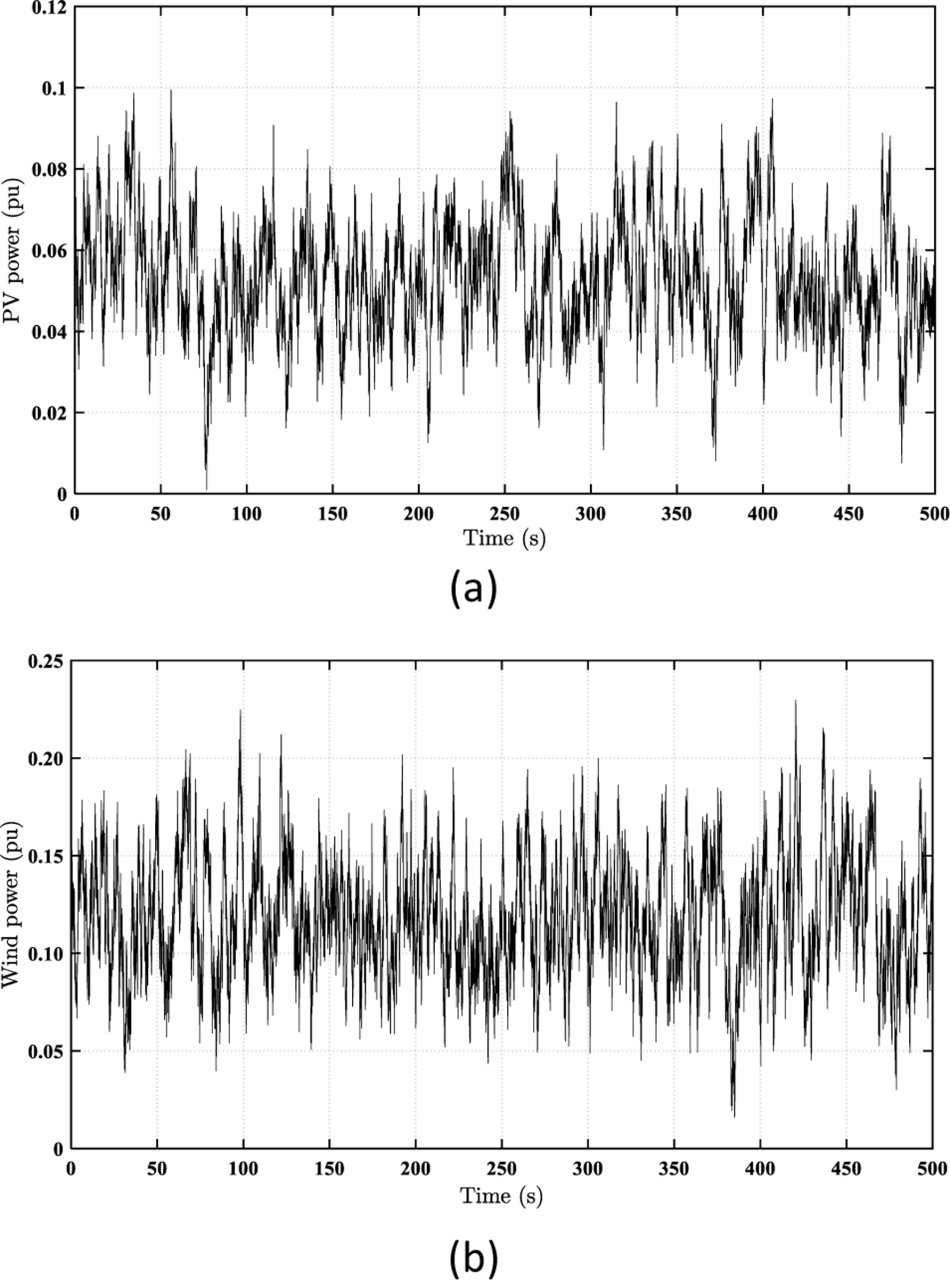
The power curves of the utilized RES. (a) PV systems (PPV); (b) WT system (PWT).

Under the various loading scenarios, Fig 6 displays the generated power (*P*_*PV*_) & (*P*_*WT*_) from the PV and WT systems, respectively. As illustrated in Fig 6, the RES has a variable nature, which has a violent effect on the FS.

### 4.1. Scenario 1

**Fig 7:**
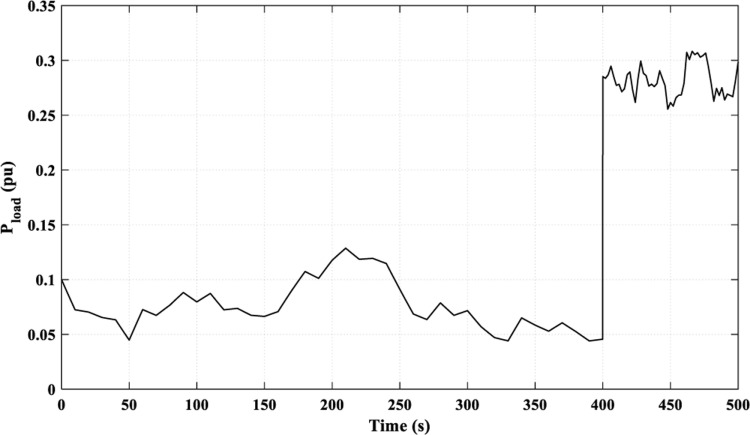
Load curve for Scenario 1.

In this scenario, Fig 7 displays the studied overall load profile. The presented overall load is a combination of domestic and industrial loads.

As presented in Fig 7, the load fluctuations ranged from 0.045 pu to 0.307 pu. According to Fig 6, the RES-generated power fluctuations ranged from 0.001 pu to 0.1 pu (for the PV system) and from 0.01 pu to 0.23 pu (for the WT system).

**Fig 8:**
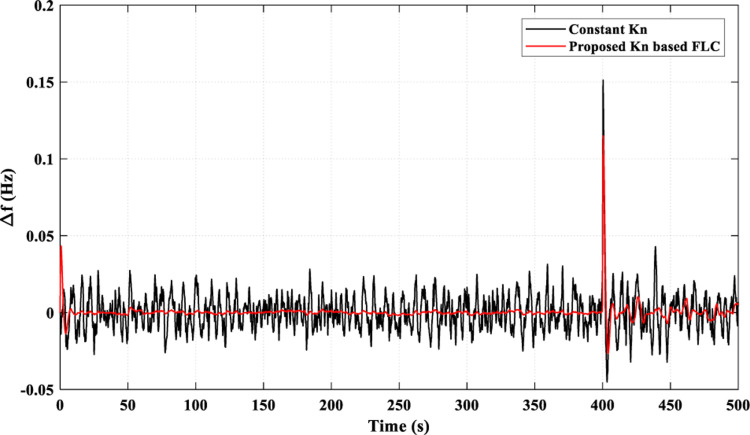
Resulting Frequency Fluctuations for Scenario 1.

Fig 8, which gives the resultant frequency fluctuations, shows that the proposed *K*_*n*_-based FLC strategy introduces a superiority over the constant *K*_*n*_ strategy, as seen at the instant of the highest fluctuation (t = 400 s), as well as the superiority of the proposed *K*_*n*_ -based FLC strategy over the constant *K*_*n*_ strategy throughout the study.

**Fig 9:**
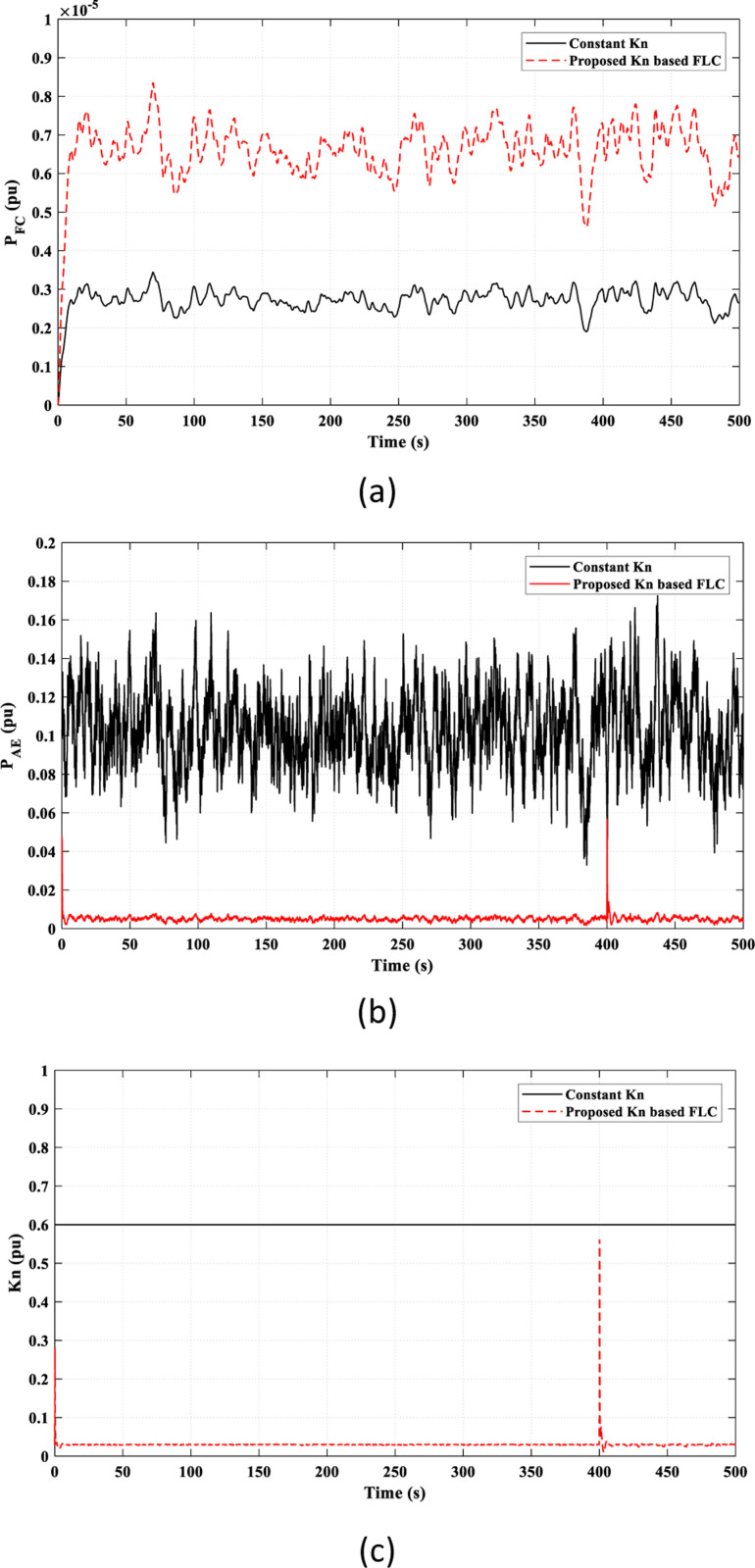
Adaptive effect of the FLC in Scenario 1 on: (a) FC power “*P*_*F*_*C* ”; (b) Input power to the AE “*P*_*AE*_”; (c) Value of “*K*_*n*_”.

The valuable effect of the *K*_*n*_-based FLC strategy provides a better adaptive behavior of the FC power (*P*_*FC*_) according to the frequency fluctuations, as shown in Fig 9 (a), which accounts for the observed superiority of the proposed *K*_*n*_-based FLC strategy over the constant *K*_*n*_ strategy in reducing frequency fluctuations, as seen in Fig 8. Also, the injected power to the AE (*P*_*AE*_) depends on the adaptive *K*_*n*_ value, as illustrated in Fig 9(b). Fig 9(c) presents the overall value of *k*_*n*_ in the study. The suggested strategy adaptively adjusts the value of *k*_*n*_ according to frequency fluctuations by the FLC. The compared work fixed the value of *k*_*n*_ as a constant value regardless of the frequency fluctuations.

**Fig 10:**
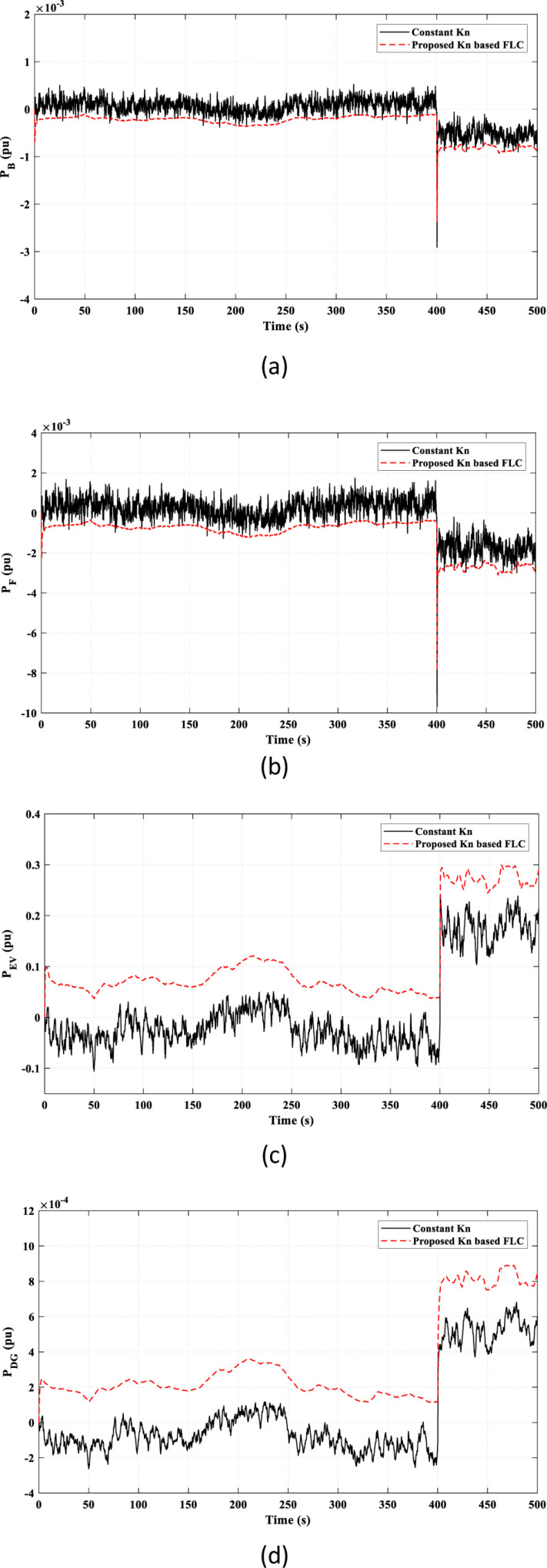
The power characteristics of the remaining elements of the studied HDGS in Scenario 1. (a) Battery “*P*_*B*_”; (b) Flywheel “*P*_*F*_”; (C) EV “*P*_*EV*_”; (d) DG “*P*_*DG*_”.

Fig 10 illustrates the power characteristics of the remaining elements in the studied HDGS. The proposed *k*_*n*_-based FLC strategy offers smoother and less fluctuating power than the constant *k*_*n*_ strategy.

Thus, the proposed strategy improved the stability and robustness of the studied HDGS during significant frequency fluctuations.

### 4.2. Scenario 2

**Fig 11:**
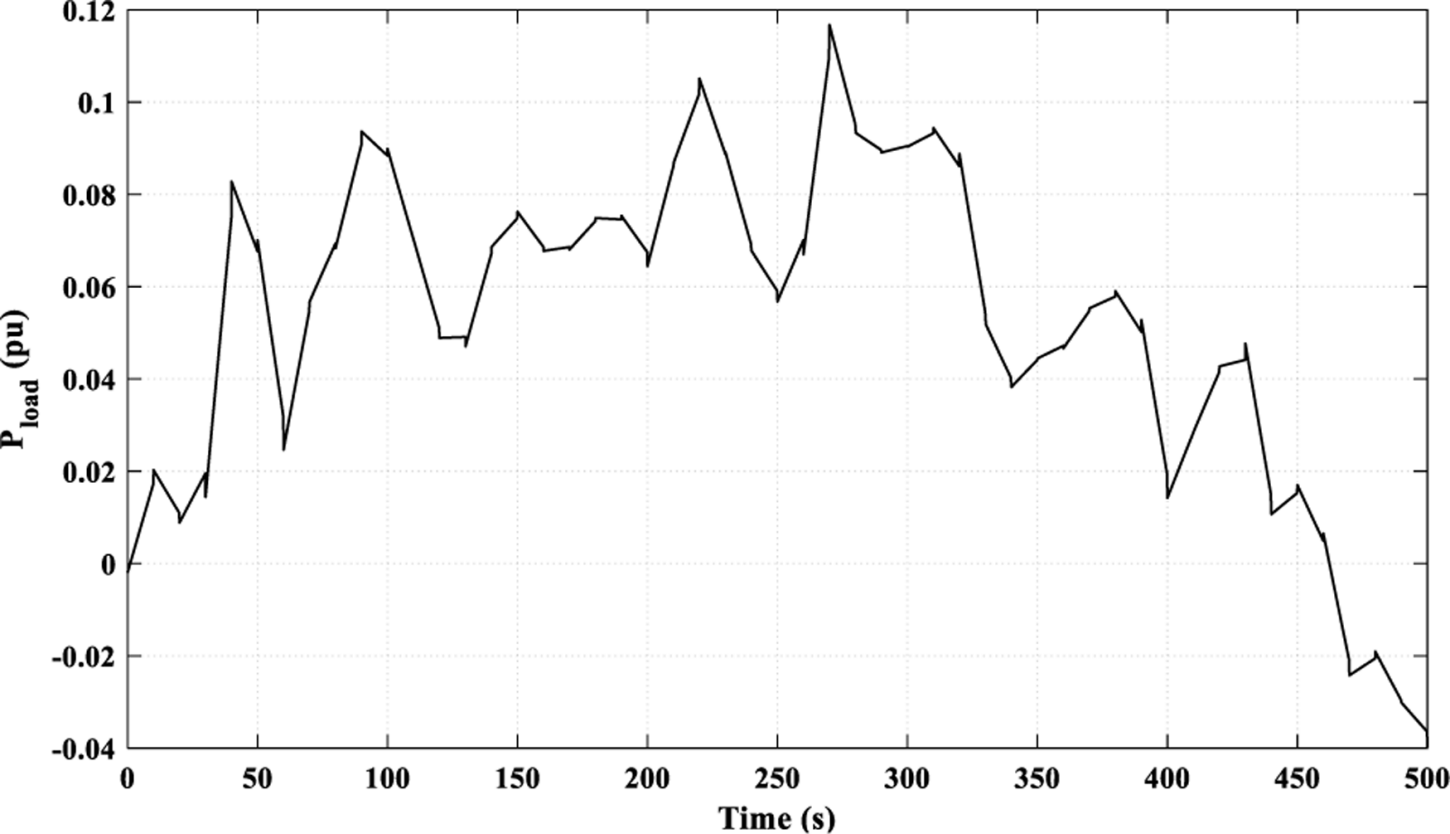
Load curve for Scenario 2.

In this scenario, the examined overall load profile is depicted in Fig 11, while the power characteristics of the RES are identical to those in Fig 6.

**Fig 12:**
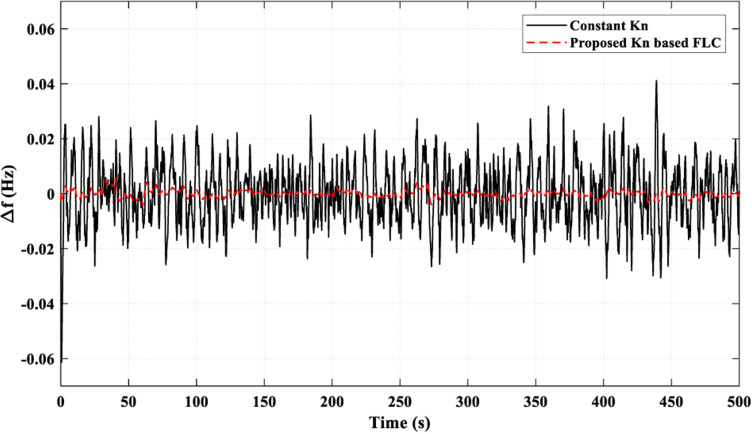
Resulting Frequency Fluctuations for Scenario 2.

Fig 12 displays the resultant frequency fluctuations. Also, the proposed *K*_*n*_-based FLC strategy introduces a superiority over the constant *K*_*n*_ strategy throughout the study.

**Fig 13:**
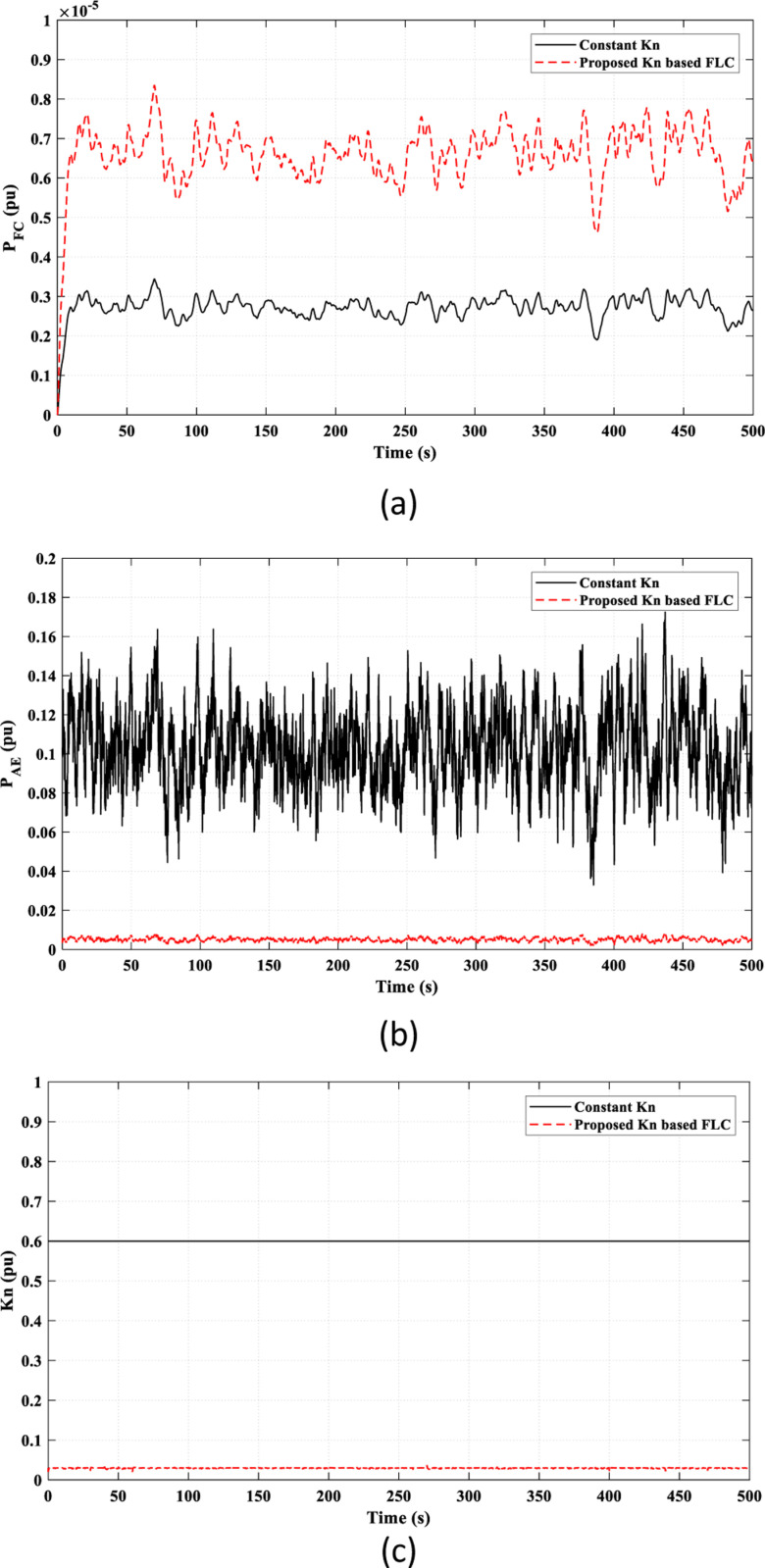
The adaptive effect of the FLC in Scenario 2 on: (a) FC power “ *P*_*FC*_ ”; (b) Input power to the AE “ *P*_*AE*_ ”; (c) The value of “*K*_*n*_”.

Fig 13 presents the effect of the proposed *K*_*n*_-based FLC strategy on the values of each *P*_*FC*_, *P*_*AE*_, and *K*_*n*_ throughout the study.

**Fig 14:**
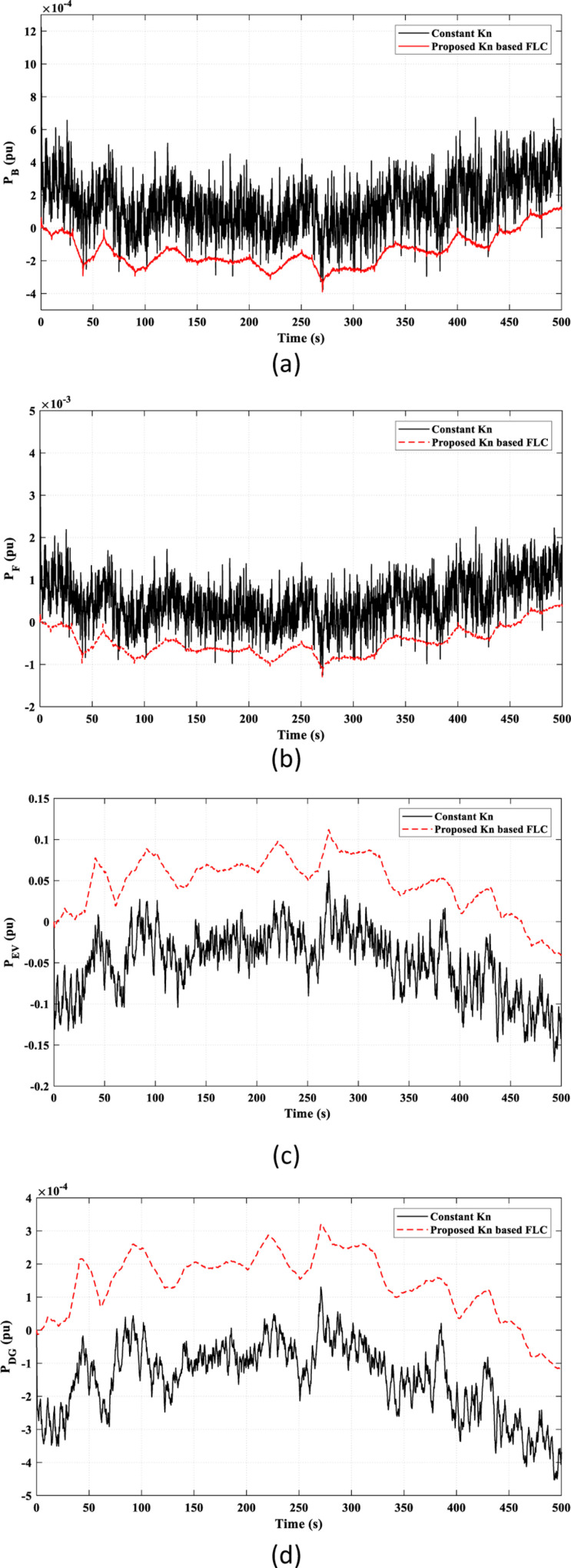
The power characteristics of the remaining elements of the studied HDGS in Scenario 2. (a) Battery “*P*_*B*_”; (b) Flywheel “*P*_*F*_”; (C) EV “*P*_*EV*_”; (d) DG “*P*_*DG*_”.

The power curves of the other elements in the HDGS are depicted in Fig 14. The proposed *K*_*n*_-based FLC strategy offers smoother and less fluctuating behavior than the constant *K*_*n*_ strategy.

### 4.3. Scenario 3

**Fig 15:**
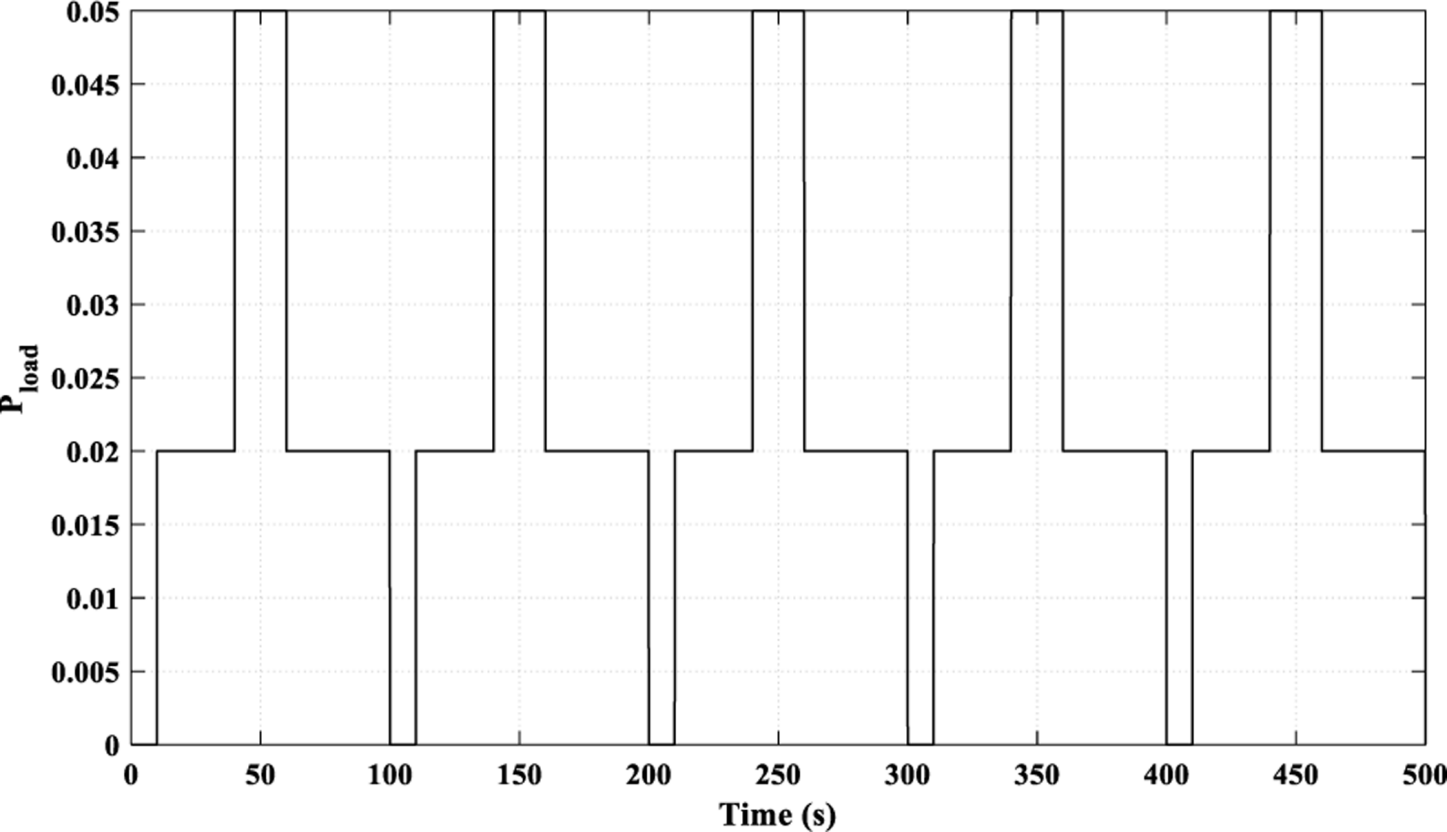
Load curve for Scenario 3.

In this scenario, the examined load is a multi-sharp fluctuating load, as demonstrated in Fig 15, while the power curves of the RES are identical to those in Fig 6.

**Fig 16:**
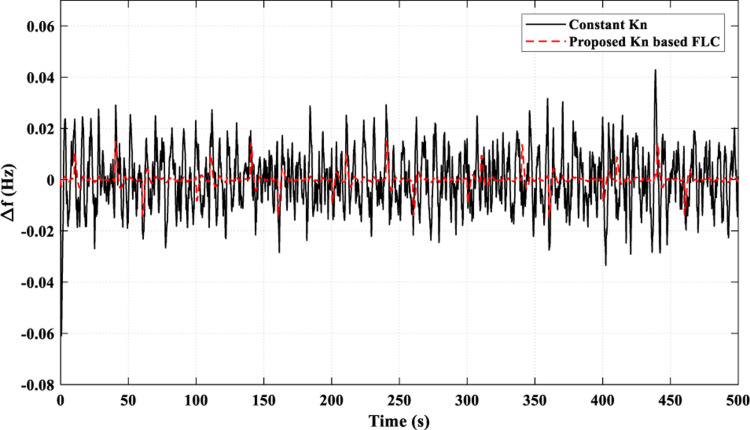
Resulting Frequency Fluctuations for Scenario 3.

**Fig 17:**
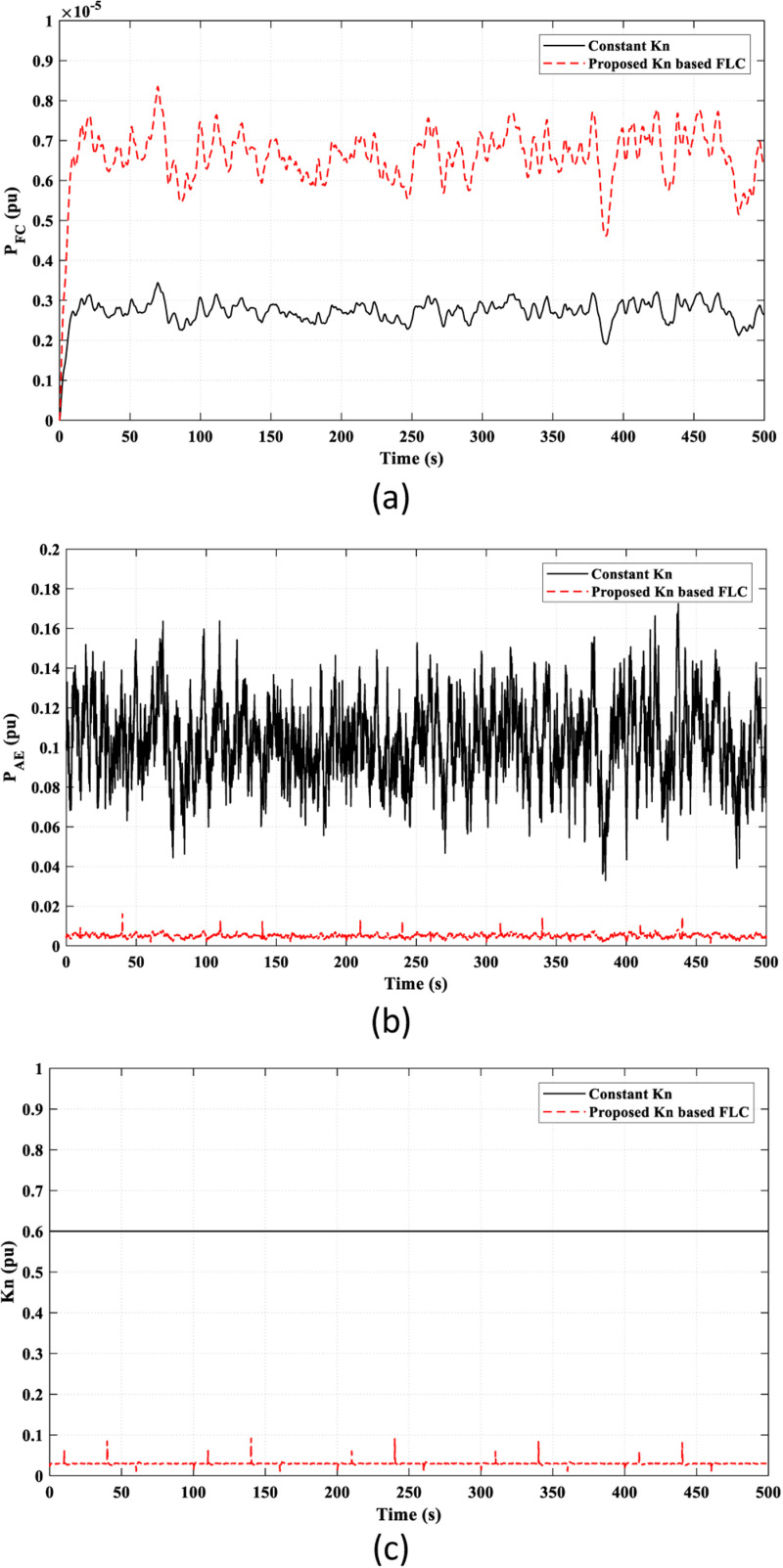
The adaptive effect of the FLC in Scenario 3 on: (a) FC power “ *P*_*FC*_ ”; (b) Input power to the AE “ *P*_*AE*_ ”; (c) The value of “*K*_*n*_”.

Fig 16 illustrates how the suggested *K*_*n*_-based FLC strategy successfully lowered the frequency fluctuations throughout the study. Fig 17 shows, throughout the study, the impact of the suggested *K*_*n*_-based FLC method on *P*_*FC*_, *P*_*AE*_, and *K*_*n*_.

**Fig 18:**
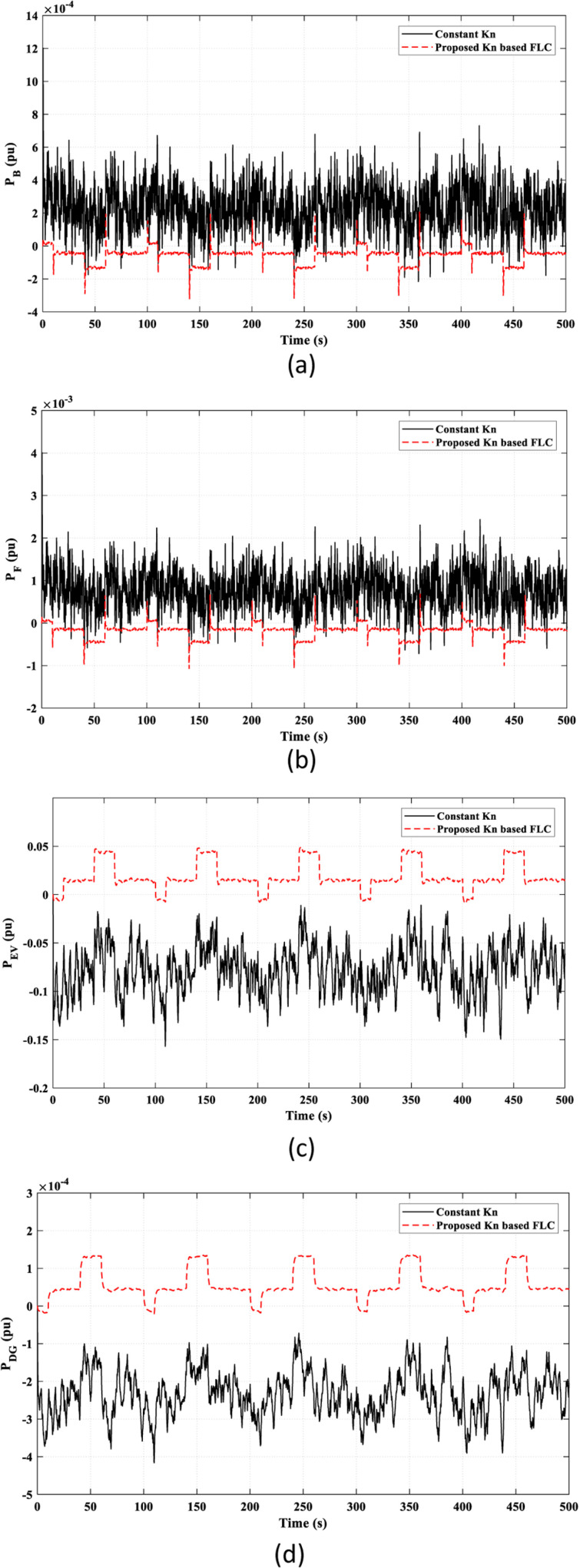
The power characteristics of the remaining elements of the studied HDGS in Scenario 3. (a) Battery “*P*_*B*_”; (b) Flywheel “*P*_*F*_”; (C) EV “*P*_*EV*_”; (d) DG “*P*_*DG*_”.

The power curves of the other elements in the HDGS are depicted in Fig 18. The proposed *K*_*n*_-based FLC strategy offers smoother and less fluctuating behavior than the constant *K*_*n*_ strategy.

### 4.4. Scenario 4

**Fig 19:**
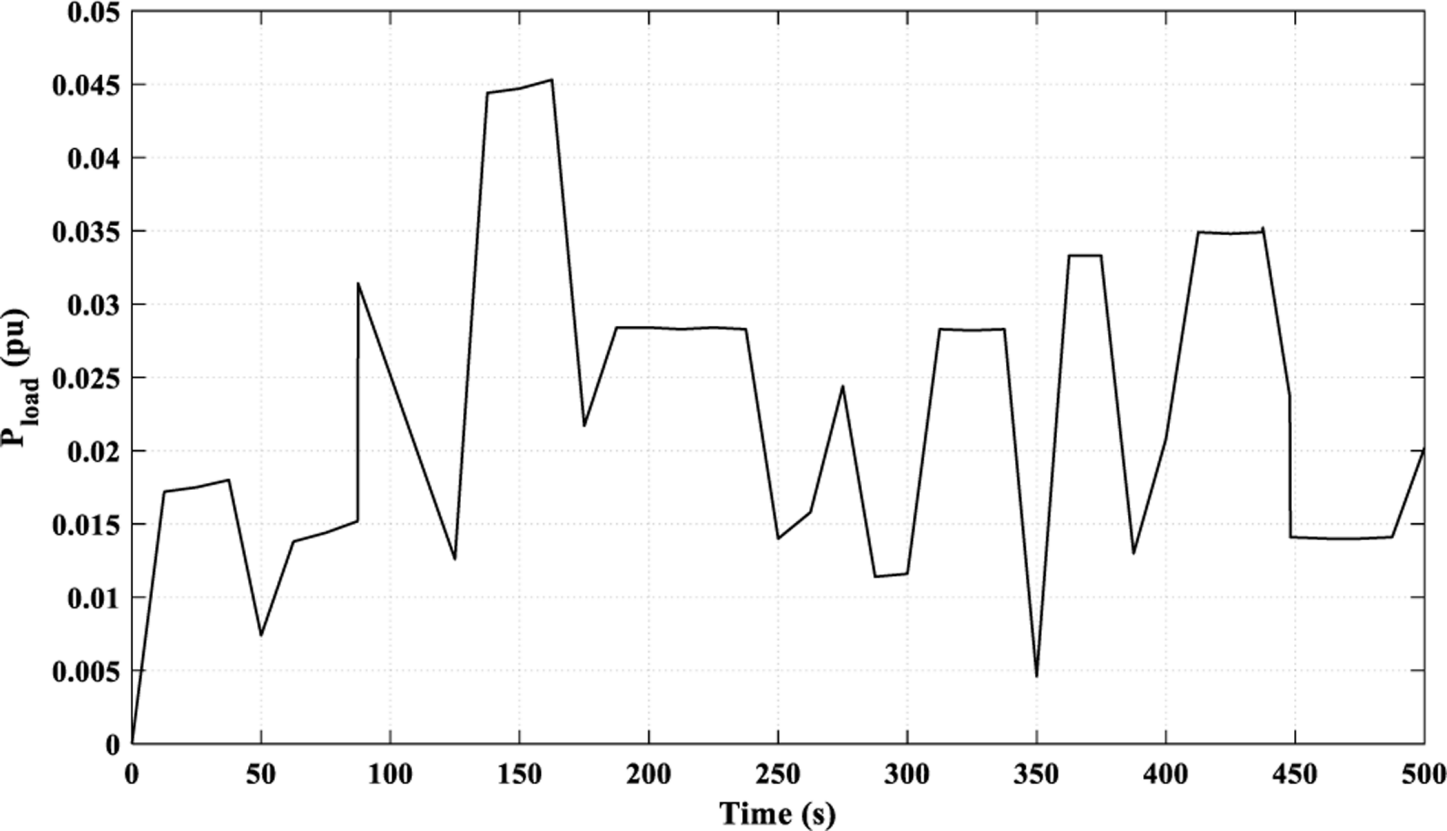
Load curve for Scenario 4.

In this scenario, the examined load is multi-fluctuating, as demonstrated in Fig 19, while the power curves of the RES are identical to those in Fig 6.

**Fig 20:**
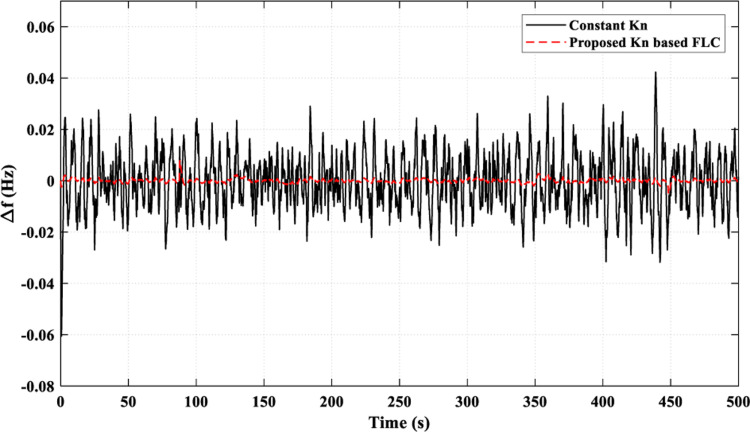
Resulting Frequency Fluctuations for Scenario 4.

**Fig 21:**
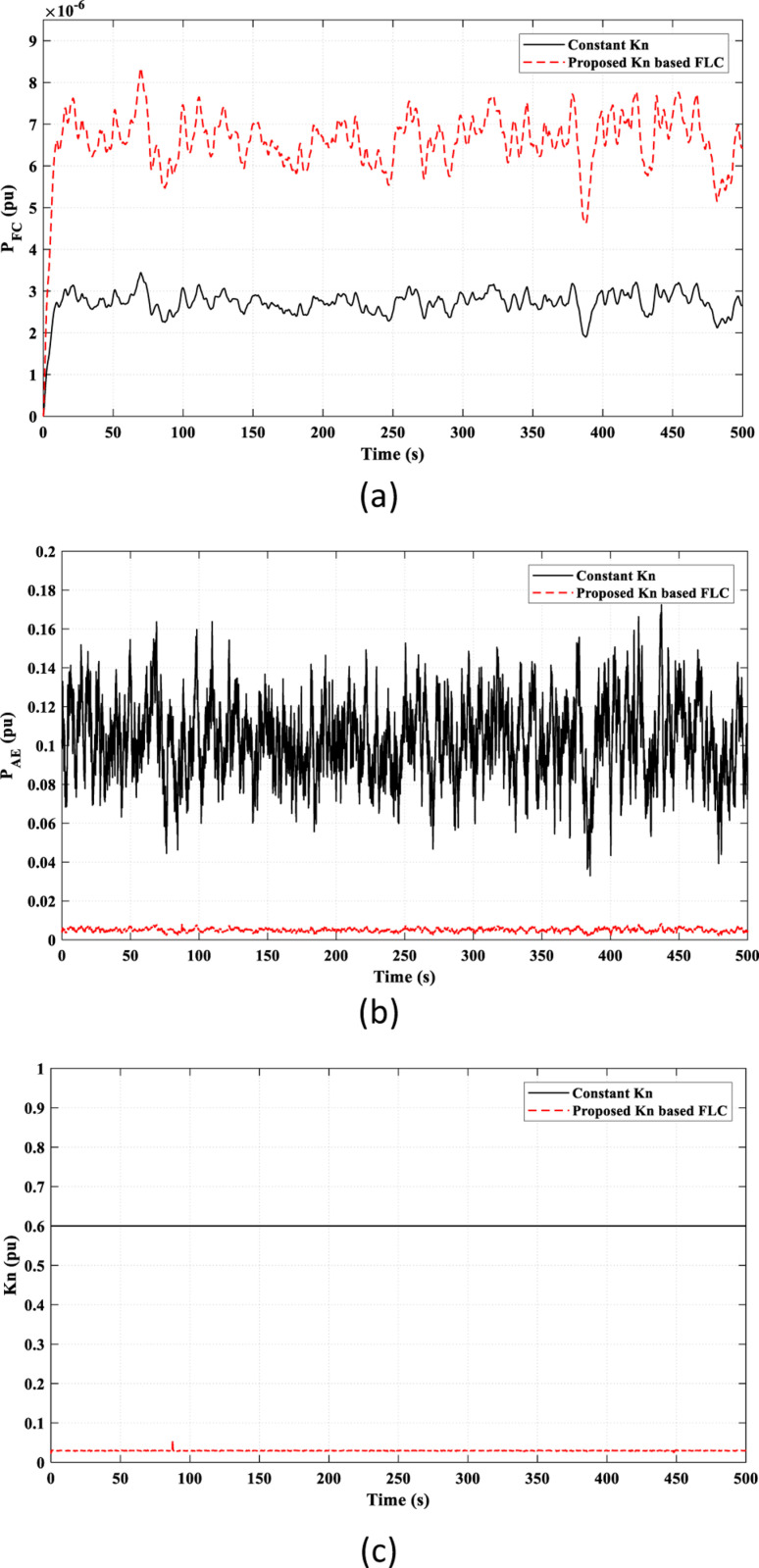
The adaptive effect of the FLC in Scenario 4 on: (a) FC power “ *P*_*FC*_ ”; (b) Input power to the AE “ *P*_*AE*_ ”; (c) The value of “*K*_*n*_”.

Fig 20 illustrates how the suggested *K*_*n*_-based FLC strategy successfully lowered the frequency fluctuations to values close to zero most of the time. While using the constant *K*_*n*_ strategy, the frequency fluctuations were limited between 0.041 pu and - 0.03 pu. This superiority of the proposed strategy over the constant *K*_*n*_ strategy is due to its ability to provide a better adaptive behavior of the FC power (*P*_*FC*_), as shown in Fig 21 (a), by controlling the injected power to the AE (*P*_*AE*_) adaptively, as illustrated in Fig 21 (b). Fig 21 (c) presents the overall values of *k*_*n*_.

**Fig 22:**
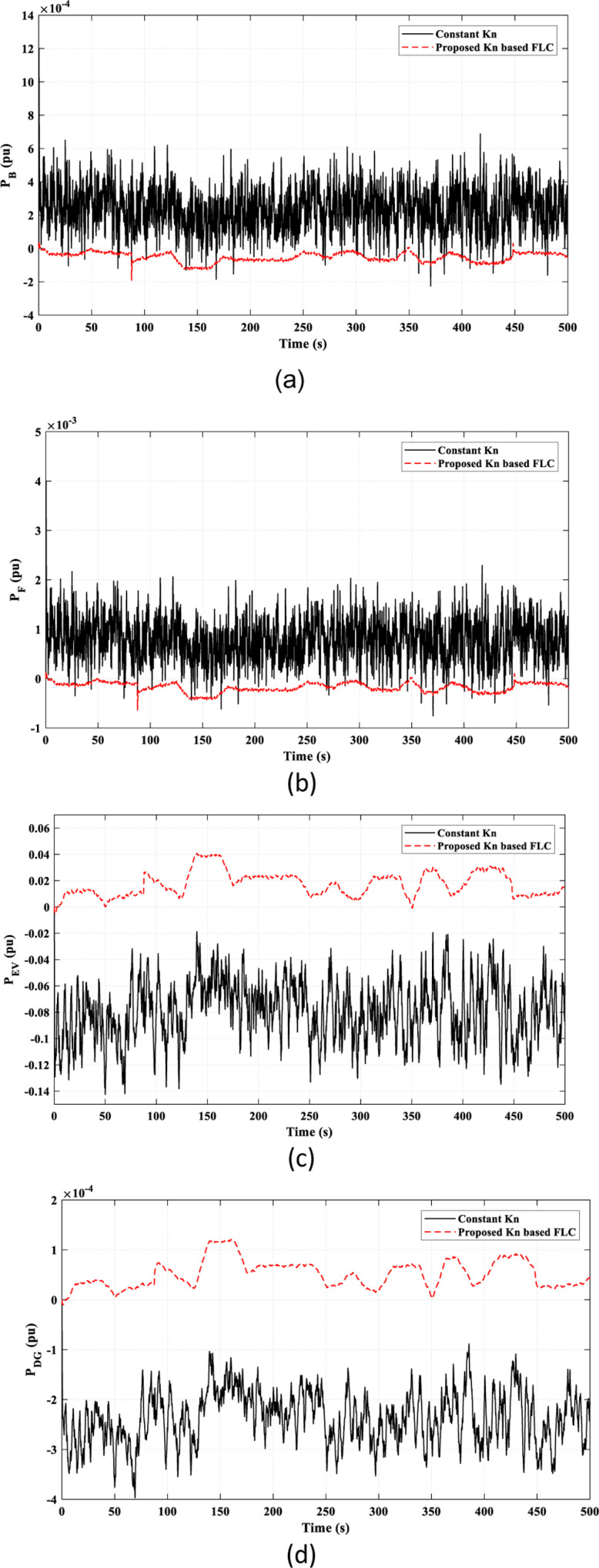
The power characteristics of the remaining elements of the studied HDGS in Scenario 4. (a) Battery “*P*_*B*_”; (b) Flywheel “*P*_*F*_”; (C) EV “*P*_*EV*_”; (d) DG “*P*_*DG*_”.

To achieve the balance in the HDGS between demand and generation, the power curves, illustrated in Fig 22, give the behavior of the remaining elements in the HDGS. Also, the proposed *K*_*n*_-based FLC strategy showed smooth and reduced fluctuations in the generated powers compared to the constant *K*_*n*_ strategy.

Table 4 presents the performance metrics of system frequency across the four applied scenarios. In Scenario 2, the system exhibits remarkable control precision with minimal overshoot of 0.005 Hz and negligible undershoot of 0.002 Hz. Conversely, Scenario 1 shows slightly higher overshoot at 0.12 Hz and undershoot at 0.02 Hz, highlighting variations in dynamic response. Scenarios 3 and 4 demonstrate intermediate behaviors with overshoot values of 0.018 Hz and 0.008 Hz, respectively, accompanied by varying degrees of undershoot.

**Table 4:** Performance of the system frequency at the different scenariors.

Metric	Scenario 1	Scenario 2	Scenario 3	Scenario 4
**Overshoot (Hz)**	0.12	0.005	0.018	0.008
**Undershoot (Hz)**	0.02	0.002	0.018	0.001

The adaptive coordination control (ACC) strategy developed in this study demonstrates practical applicability, especially for hybrid distributed generation systems (HDGS) with high renewable energy penetration and variable load conditions. Utilizing fuzzy logic control (FLC), the ACC facilitates rapid, real-time decision-making, effectively managing dynamic renewable fluctuations and sudden load variations. Its simplicity and computational efficiency enable straightforward implementation. The dynamic adjustment of the power-sharing factor (Kn) ensures adaptability across diverse operational scenarios, enhancing effectiveness in environments characterized by intermittent renewable resources like solar PV and wind turbines. Moreover, ACC exhibits strong scalability, easily accommodating additional renewable sources or advanced energy storage technologies without significant reconfiguration. The strategy has proven robust under challenging conditions, including severe frequency deviations and rapid load changes, thereby maintaining system stability and reliability during disturbances. Nevertheless, practical implementation requires careful consideration of challenges such as sensor accuracy, communication latency, parameter tuning, and data reliability to ensure optimal performance.

Finally, based on the results obtained from different scenarios, the adaptive coordination control (ACC) solution demonstrates strong scalability due to its adaptive fuzzy logic control (FLC), which dynamically adjusts the power-sharing factor (Kn) according to frequency deviations. This adaptability allows effective management of additional renewable energy sources, handling increased variability without significant performance degradation. Moreover, integrating extra renewable energy sources introduces fluctuations that the ACC compensates for by optimally allocating surplus renewable energy to hydrogen production through the aqua electrolyzer (AE), thus maintaining frequency stability. Additionally, expanding energy storage capacity—such as fuel cells, batteries, flywheels, and EVs—further enhances system flexibility. The ACC efficiently utilizes these additional storage resources to buffer and balance the greater renewable generation, ensuring robust frequency stabilization.

## 5. Conclusions

An adaptive coordination control strategy for enhancing the frequency stability of an HDGS with RESs was presented in this work. The studied HDGS included different power sources like PV, WT, AE, FC, DG, EV, BESS, and FESS. In the HDGS, frequency fluctuations resulted due to the intermittent nature of the RES and the fluctuating demand on the other side. To stabilize the system frequency under various loading scenarios, the proposed CCS used the FLC to control the ratio of directed power from the RESs to feed the AE, thereby controlling the FC power. The proposed CCS was tested under four scenarios with different load profiles, and its robustness and effectiveness were validated through comparison with a prior study. The significant findings may be summed up as follows:

- Under varying generation and load situations, the suggested CCS effectively reduced frequency variations.
- All assessment scenarios saw the frequency kept at a nearly constant value by the suggested CCS.
- The suggested CCS is superior to the compared strategy at lowering frequency deviation.
- The proposed CCS adaptively optimized the power-sharing between RESs and FCs via AE.

While the current manuscript focuses primarily on comparing the adaptive fuzzy logic-based ACC to traditional fixed-value strategies, comparing the proposed approach with other advanced control techniques, such as model predictive control (MPC) or deep reinforcement learning (DRL), would further highlight our approach’s strengths and potential limitations. So, these comparisons will be explored comprehensively in future research extensions.
